# Economic factors influencing the empowerment of Peruvian women

**DOI:** 10.12688/f1000research.149817.2

**Published:** 2025-03-21

**Authors:** Elena Jesús Alvarado Cáceres, Enaidy Reynosa Navarro, Lindon Vela Meléndez

**Affiliations:** 1 Universidad Cesar Vallejo, Av. Alfredo Mendiola 6232, Los Olivos, 15314, Peru; 2 Universidad César Vallejo, Av. Larco 1770, Trujillo, 13001, Peru; 3 Universidad Nacional Pedro Ruíz Gallo, Juan XXIII 391,, Lambayeque, 14013, Peru

**Keywords:** Women's Empowerment; Financial Capacity; Legal Framework; Economic Independence; Potential Customers; Access to Financing

## Abstract

**Objective:**

To determine whether economic factors are crucial in empowering women, guiding them towards growth and development opportunities, achieving empowerment, and contributing to two sustainable development goals of the 2030 development agenda: ending poverty and achieving gender equality.

**Methodology:**

The research was foundational, with a phenomenological and hermeneutic design. The applied technique was in-depth interviews with 12 women who had started a business within the last five years in a region of Peru.

**Results:**

It is evident that economic factors are decisive in business experiences and decisions, highlighting the necessity of having contingency funds to prevent operational impacts. Through entrepreneurship, women achieved economic independence, enabling them to support their families and impacting their empowerment. It concludes that to promote economic opportunity equality, addressing financing needs, encouraging economic independence, strengthening family empowerment, improving customer management, and facilitating access to government funds are essential.

**Conclusions:**

The narrative of the participants provides a solid foundation for designing specific policies and support programs that boost the economic empowerment of women entrepreneurs and encourage their active participation in the business sphere.

## 1. Introduction

Women’s empowerment has been identified as a fundamental pillar for sustainable economic growth and social development. ^1^ In this context, economic freedom emerges as a critical factor enabling women to make economic decisions independently, thereby contributing to their personal and professional empowerment. However, in developing countries like Pakistan, women continue to face numerous disadvantages that limit their economic participation and their ability to make free decisions in the economic sphere. In this sense, relevant studies have been focusing on analyzing how economic freedom can foster women’s empowerment beyond the cultural, social, and legal barriers that, in one way or another, restrict female participation in the economy. ^1– 4^

Moreover, it’s essential to highlight that the aforementioned obstacles hinder women’s full economic freedom, including limitations on women’s mobility, the decision to work, equal pay, legal barriers to marriage, working after childbirth, registering a business, property division, and regulating women’s pensions; among other limitations such as the lack of education and access to technology and its latest advancements. In this regard, access to social networks and media has also been identified as a catalyst for women’s economic empowerment, suggesting a positive correlation between women’s economic empowerment and their demographic, social, and economic characteristics. ^1^

Scientific publications highlight that, in developing countries like Ethiopia, significant challenges are faced, including high population growth rates and limited opportunities for women in education and employment. These challenges underscore the importance of focusing on women’s empowerment through access to resources and agricultural inputs as key strategies to overcome economic and social barriers. ^1^ Under this premise, social norms and restricted access to education and employment opportunities represent significant obstacles to women’s economic empowerment, particularly in rural areas. Factors such as access to and control over assets, agricultural inputs, credit, extension services, and social networks have been shown to have a positive impact on women’s economic empowerment. ^1^ However, despite growing attention to the socioeconomic factors influencing women’s empowerment, ^5, 6^ there is a pressing need to promote sustained efforts for their empowerment over time.

To foster the economic empowerment of women entrepreneurs, it is crucial, in addition to a strong motivation to achieve ambitious goals in challenging contexts, as previously mentioned, that they have support facilitating the articulation of their objectives. Recent research underscores that support from entities, such as the government, plays a significant role in women’s economic empowerment. ^2^ This backing can take various forms, including financial education, which is fundamental for women’s economic empowerment. This is because it enables them to make financial decisions based on solid information and manage their economic resources effectively.

Ultimately, female empowerment aims not only to improve women’s income and living standards but also to enhance their decision-making capacity. However, an important fact must be considered: women’s vulnerability often stems from their limited participation in decision-making processes and their restricted access to essential resources, ^4^ which may include access to education, healthcare, employment opportunities, and involvement in decision-making processes. To address these issues, it is crucial to consider the livelihood options available to women. Policies promoting favorable conditions, such as access to relevant information and reduced interest rates, are vital to support women’s empowerment and encourage their economic independence. ^4^

In Peru, there is a Legal Framework and Public Policies with solid foundations aimed at supporting female entrepreneurship and promoting the economic empowerment of women. Law No. 31828, the Young Entrepreneur Law, aims to establish a regulatory framework to generate employment opportunities for youth that successfully contribute to the country’s development within a social market economy ^7^; it creates a favorable environment for young entrepreneurs, including young women, by facilitating the establishment and registration of businesses and offering tax benefits that encourage hiring.

Additionally, the National Women Entrepreneurs Strategy, established through Supreme Decree No. 012-2022-MIMP, aims to “promote and develop the economic autonomy of women in their diversity to achieve the reduction of structural gender gaps and the enjoyment of a sustainable, dignified life with full well-being”. ^8^ This comprehensive strategy focuses efforts on creating conditions that allow women to overcome the historical and structural barriers that limit their economic empowerment.

On the other hand, Law No. 31168, which promotes the empowerment of rural and indigenous women, aims to “strengthen, through affirmative actions, the empowerment, equality of opportunities, and comprehensive development of rural and indigenous women, enhancing their economic, cultural, social autonomy, through training and productive financing”, ^9^ constitutes a pillar in the fight for equality by establishing positive action measures and promoting access to productive and financial resources, especially aimed at women in vulnerable situations.

Furthermore, Supreme Decree No. 008-2019-MIMP is another fundamental tool aimed at ensuring the exercise of women’s economic and social rights, promoting access to productive resources, and improving conditions for their formal labor insertion. ^10^ This policy strengthens the existing legal framework, driving towards a more inclusive and equitable society.

Despite this progressive legal framework and policies designed to support female entrepreneurship and economic empowerment, significant challenges persist in Peru that limit the full realization of these objectives. Women entrepreneurs still face structural obstacles, such as insufficient financial capacity, lack of knowledge of the legal framework and the norms and procedures for formulating their ventures, lack of economic independence, difficulties in attracting and retaining potential customers, as well as limitations in accessing support funds (both national and international) for entrepreneurship, among others. These barriers underline the need for effective and sustained implementation of existing laws and policies, as well as the importance of adopting additional measures to address residual inequalities and foster a truly empowering environment for women in the business sphere.

In this regard, the population of women entrepreneurs, compared to men in the northern region of Peru, is higher in percentage terms: La Libertad region (51.20%), Cajamarca region (56.60%), Piura region (55.10%), and Tumbes region (54,50%). ^11^ In the second quarter of 2023, women in the northern region of Peru, as sole proprietors, led 55.7% of formal business creation. Specifically, in the retail sector, 63.9% of newly established business units were managed by women. ^11^

The situation of northern women entrepreneurs was marked by historical marginalization and the limited recognition of their contributions, despite their participation in political, economic, and social processes. These women faced structural restrictions and discrimination in spheres of power and knowledge. ^12^ However, the triangulation of information revealed that they organized collective savings systems and rights defense networks. Additionally, they established alliances with producers and communities to drive production, generate benefits, transform local practices, create a positive impact on the regional economy, foster civic engagement, and promote positive changes in their communities, laying the foundation for sustainable economic development. ^12– 14^

The participants in this study had higher representation in beauty salons (73.8%), human health care activities (67.5%), food and beverage service activities (62.6%), and retail trade (61.3%). Encouragingly, regions such as Piura (55.1%), Tumbes (54.4%), Lambayeque (51.9%), and La Libertad (51.2%) experienced greater growth compared to men in terms of sole proprietorship representation. ^11^ However, it is concerning that, except for Cajamarca (47.4%), the other regions lead the list of business closures relative to men: La Libertad (52.0%), Lambayeque (54.4%), Piura (56.0%), and Tumbes (57.4%). ^11^

Gender inequality in Peru is evident through various gaps in labor participation, wages, and domestic workload. Only 51% of working-age women actively participate in the labor market, facing a 25% wage gap. Additionally, 41% of women leave their jobs after the birth of their first child, a trend that persists in the long term. In terms of poverty, warnings have been issued since 2019 that 51.6% of women lived in monetary poverty, while extreme poverty affected 52.2% of them. Unlike men, rural women experience higher levels of poverty, further exacerbating economic disparities. ^15^

This research directly contributes to the following Sustainable Development Goals (SDGs):

SDG 1: No Poverty. This goal aims to eradicate poverty in all its forms and dimensions. Additionally, SDG 5: Gender Equality. This goal seeks to achieve gender equality and empower all women and girls. The study aligns with the following targets: 1) Target 5.4: Recognize and value unpaid care and domestic work. 2) Target 5.5: Ensure women’s full and effective participation in the economy.

The twelve Peruvian women entrepreneurs participated in various economic sectors, including trade, agribusiness, fishing, handicrafts and textiles, and gastronomy. Each sector presented specific challenges: in agribusiness and fishing, dependency on climatic factors and limited access to technology and financing; in handicrafts and textiles, restricted access to broader markets and reliance on tourist demand; and in gastronomy, high competition and difficulties in business formalization. Although sectoral nuances existed, structural and cultural barriers were found to universally limit empowerment. ^16^

The objective of this study focused on determining whether economic factors are crucial in women’s empowerment. As specific objectives, it sought to evaluate how the economic needs of female-led ventures lead to the creation of a contingency fund, determine how the establishment of businesses has generated economic independence for women entrepreneurs, identify how women entrepreneurs have achieved family empowerment through economic success, analyze the importance of managing customers in a venture, determine the significance of formality in regulatory compliance in female-led enterprises, and assess whether women entrepreneurs have access to government funds.

## 2. Methods

### 2.1 Design

This research is framed within a naturalistic paradigm and follows a qualitative approach, which allows understanding to what extent economic factors are determinants in the empowerment of Peruvian women based on their perceptions and meanings. Additionally, it features a hermeneutic phenomenological design, “aimed at the description and interpretation of the fundamental structures of lived experience” ^17^; under this design, it was possible to explore and understand the lived experiences of the participating women entrepreneurs, based on their narrative derived from their experiences, their reality, and their entrepreneurial ecosystem.

### 2.2 Participants

The participants included in the study were 12 entrepreneurs from the northern region of Peru who, at the time of the research, owned a medium or small business, which they had been developing for a minimum period of five years. All were female, with ages ranging from 25 to 60 years, and an average age of 35.33 years. All expressed their willingness to participate in the study in a friendly and cordial manner.

### 2.3 Research team, characteristics, and flexibility

The research team, comprised of academics from two Peruvian universities, contributed extensive knowledge on economic empowerment, enriching the study with their academic and professional diversity. The applied hermeneutic phenomenological methodology allowed for the establishment of meaningful relationships with the participants, promoting a trustful environment where they freely shared their experiences. Aware of how our experiences and perspectives could influence the interpretation of the data, we adopted an “epoché” attitude, striving to understand the participants’ narratives without prejudice. This interaction between our characteristics and the study was key in shaping the approach, methodology, and interpretation of results, allowing us to identify patterns and emerging themes that enriched our findings. The adopted approach and close relationship with the participants captured the complexity of women’s economic empowerment in Peru, facilitating the transferability of the research.

### 2.4 Context

The research was conducted in the northern region of Peru, an area characterized by a dynamic entrepreneurial ecosystem reflecting similar trends across the country. However, in this area, female entrepreneurship has begun to stand out significantly in recent years, challenging traditional barriers and contributing to the economic and social fabric. The choice of this region as the study site is due to its relevance in the country’s entrepreneurial landscape and the opportunity to explore the specific impact of economic factors on women’s empowerment in a context where women entrepreneurs are emerging as key actors for sustainable development. The study focuses on analyzing whether economic factors, such as access to financing and economic independence, act as catalysts for women’s empowerment, guiding them towards opportunities for growth and personal and professional development. Additionally, this research corroborates the need to better understand how female entrepreneurship can contribute to achieving key sustainable development goals, particularly ending poverty (Goal 1) and achieving gender equality (Goal 5), in the 2030 development agenda. ^18^

### 2.5 Sampling strategy

The participant selection methodology for this research was centered on purposeful sampling targeting women entrepreneurs who had launched their projects within the last five years in the northern region of Peru. This approach was specifically designed to capture recent and relevant experiences that reflect both the challenges and opportunities within the realm of female entrepreneurship. The intention was twofold: to align the study with its core objectives and to deepen the understanding of how economic factors impact women’s empowerment. Twelve women participants were selected, a number deemed optimal for achieving data saturation. This decision was based on the observation that additional interviews were not yielding new themes or significant data. Continuous analysis and review of the interviews, facilitated by the use of ATLAS.ti software, allowed for the identification of recurring themes and patterns, as well as the confirmation of key categories and subcategories. These observations indicated that a comprehensive understanding of the participants’ narrated experiences had been achieved, suggesting that the sampling process had reached its saturation point and, therefore, the number of participants was sufficient for the purposes of the study.

### 2.6 Ethical considerations

In this study, the respect, integrity, and protection of participants’ rights were ensured. Before conducting the in-depth interviews, written informed consent was obtained from all participating entrepreneurs. This consent process not only ensured that participants were fully informed about the nature and purpose of the study but also protected their rights and confidentiality. They were fully informed about the study’s objectives, their role in it, and how their personal information would be handled and protected. Confidentiality and anonymity of the participants were guaranteed during the processing of their data and testimonies, avoiding any form of harm or discrimination. ^19– 21^ Additionally, special care was taken in the sensitive handling of topics that could evoke intense or traumatic personal experiences, providing a safe and supportive space during the interviews. It is worth noting that this study aligns with the sustainable development goals of the 2030 Agenda, specifically regarding the eradication of poverty and gender equality. ^18^

The research project titled “ Personal, Economic, and Social Factors Influencing Women’s Empowerment,” was approved by Vice-Chancellor for Research Resolution N° 442-2022-VI-UCV. ^22^ Subsequently, the Ethics Committee in Research of the School of Administration at Universidad César Vallejo reviewed the project under the review code 2024-1-36 and granted a favorable opinion on January 26, 2024. The research was conducted in accordance with the ethical principles outlined in the Declaration of Helsinki, ensuring informed consent from all participants and guaranteeing the confidentiality and anonymity of the collected information.

### 2.7 Data collection techniques

An in-depth interview was conducted, whose base code was Female Entrepreneurship, from which the study’s central category (Economic Factors) was derived, divided into five subcategories: Financial Capacity, Legal Framework, Economic Independence, Potential Customers, and Access to National and International Funds. This allowed participants to express their experiences, perceptions, and emotions openly, yet guided by a set of previously prepared questions that steered the conversation towards the study’s topics of interest. Given the wide geographic distribution of the entrepreneurs, the interviews were recorded via Zoom with an approximate duration of 60 minutes per interviewee. The applied interview was subjected to content validity by five experts in the field – as recommended in the specialized literature. ^23– 25^

### 2.8 Data processing and analysis

The interview results were transcribed and analyzed using the content analysis technique, aiming to identify patterns, themes, and emerging categories that reflect the experiences and perceptions of women entrepreneurs. Additionally, a hermeneutic interpretation of the participants’ narratives was conducted to understand the deep meanings of the experiences narrated by the participants. For the analysis and processing of all the information, the qualitative analysis software ATLAS.ti was used, enabling coding, analysis, visualization, online collaboration, and flexibility in managing the obtained data. ^26^

## 3. Results

Next, we explore qualitative categories and subcategories structured from interviews with women entrepreneurs, focusing on how these economic factors influence their experiences and business decisions. Through the narratives of these women, we examine how the need to have funds for contingencies has affected their operations, how they have achieved economic independence through entrepreneurship, how family support has impacted their empowerment, how they identify and relate to their potential customers, and what their experiences are with government entrepreneurship support programs.

**Figure 1.**
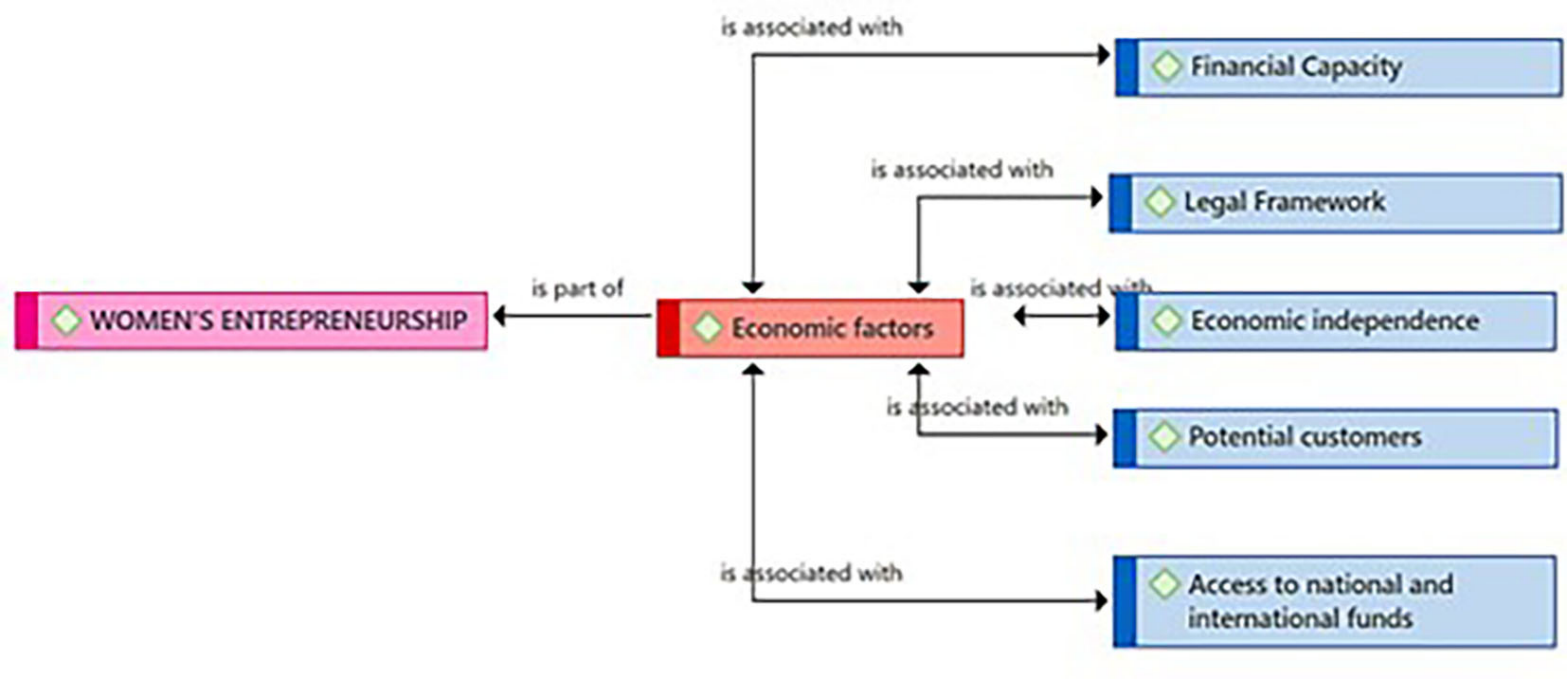
Economic Factors category.

Figure 1 presents the Economic Factors category as a component resulting from the coding process of the central theme (Female Entrepreneurship). According to the entrepreneurs, these economic factors are linked to the Financial Capacity subcategory, necessary for managing those financial resources essential for the success of female entrepreneurship. The practitioners consider the existence of a Legal Framework with the security that is felt when an enterprise is not only successful but also formal. Likewise, they identified Economic Independence as another key related subcategory. Regarding the Potential Customers subcategory, they consider it also connected to Economic Factors. Finally, the entrepreneurs consider that the subcategory Access to national and international funds represents a need that goes beyond local borders for the growth and expansion of Female Entrepreneurship.

**Figure 2.**
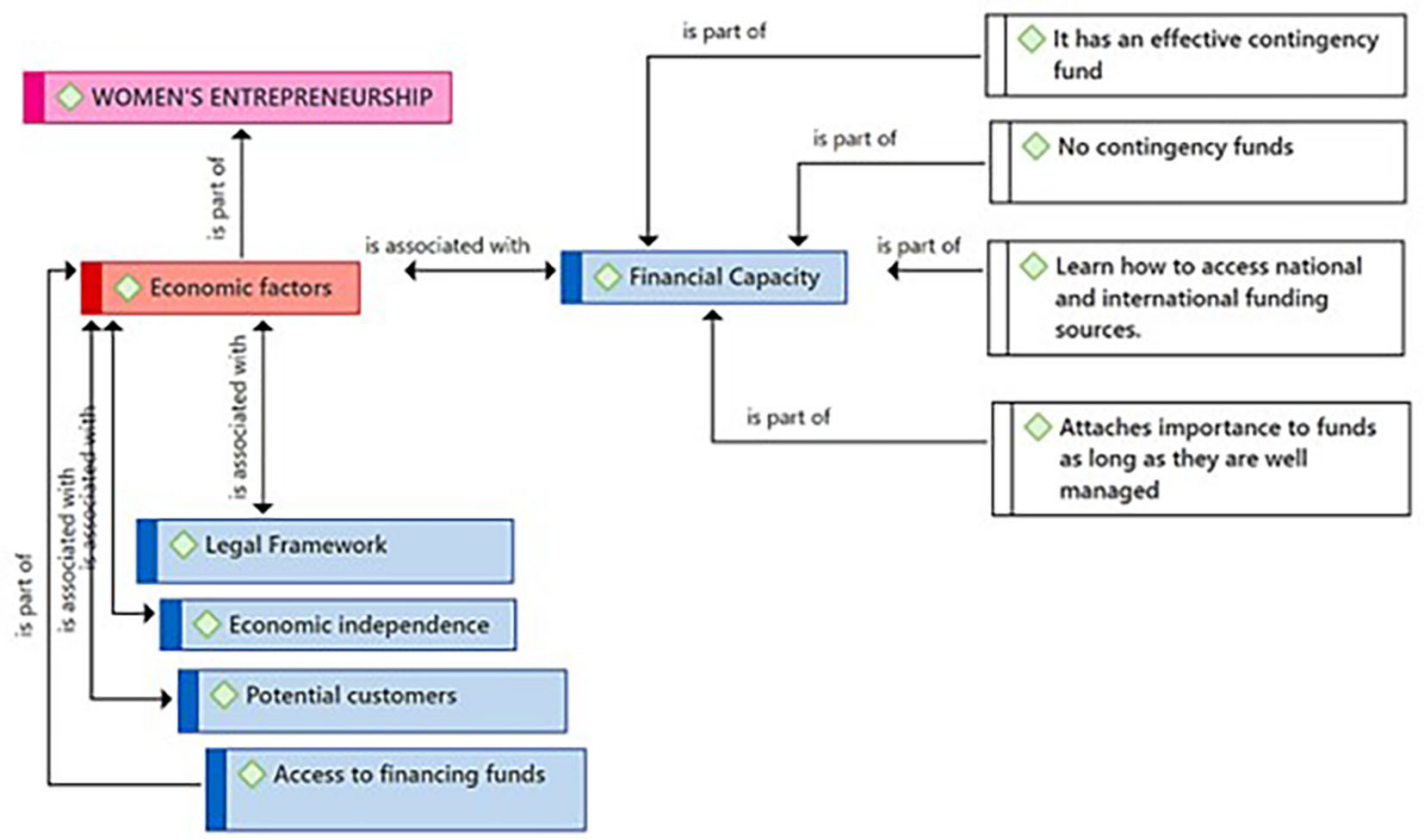
Financial Capacity subcategory.

Figure 2 highlights Financial Capacity as a critical subcategory in the context of female entrepreneurship, as perceived by the participants. From their point of view, financial capacity encompasses fundamental elements such as: having an effective fund to face contingencies; the awareness and/or situation of not having funds for contingencies, highlighting a concern for vulnerability to unforeseen events; knowledge about how to access national and international financing sources; and the importance attributed to proper fund management, reflecting a focus on responsibility and efficiency in financial resource management.

**Figure 3.**
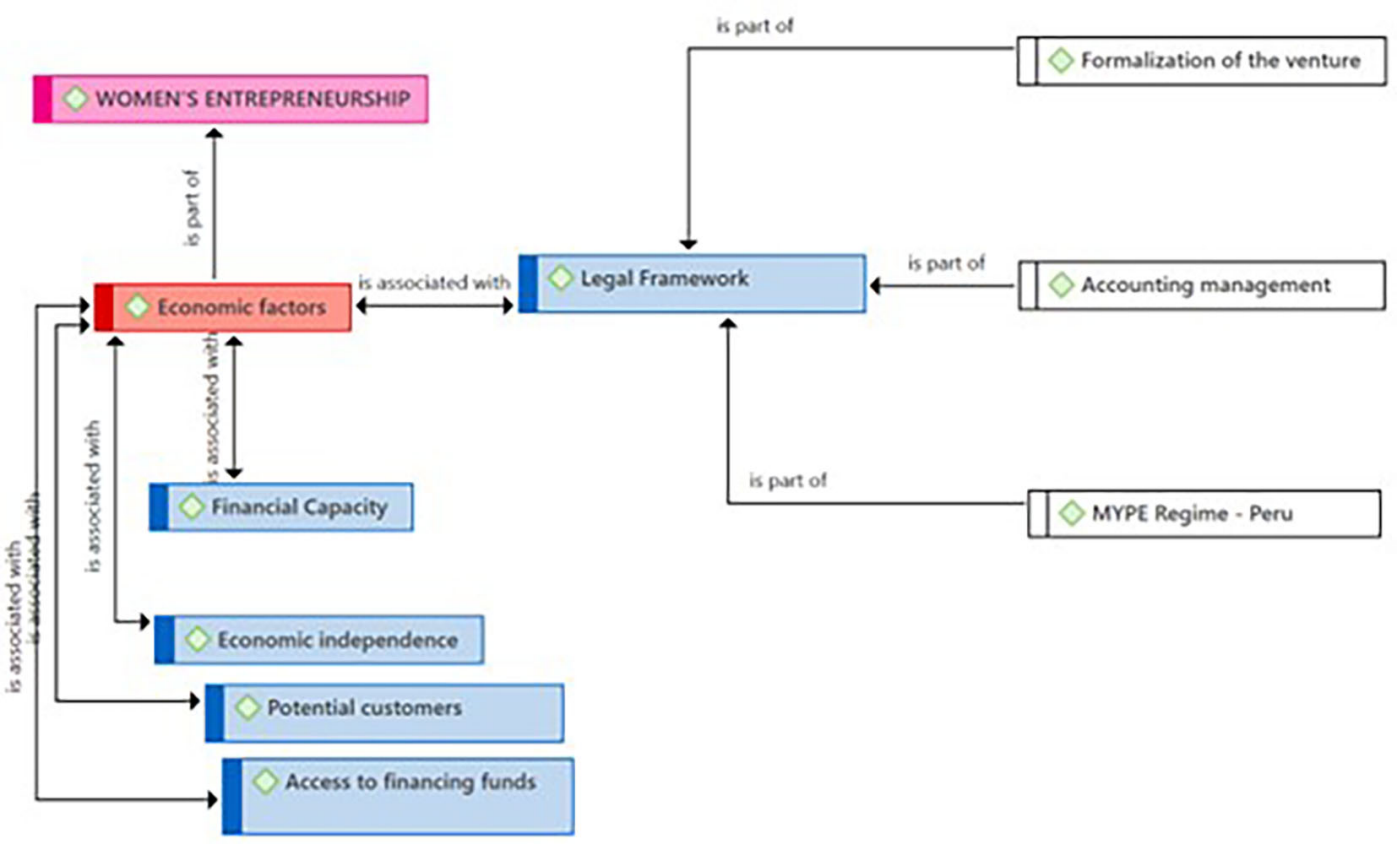
Legal Framework subcategory.

In Figure 3, according to the entrepreneurs, the Legal Framework subcategory, in addition to being an essential component for entrepreneurship, is fundamental for the formalization of the venture. Regarding accounting management, they maintained that having rigorous accounting aligned with legal norms is an essential element for achieving transparency and financial health of their enterprises. As for the “Régimen MYPE-Peru” issue, in some cases, the entrepreneurs point out the need to join this regime, while in others, they emphasize that remaining within this regime is vital for the optimal functioning and growth of their businesses within the local economy.

**Figure 4.**
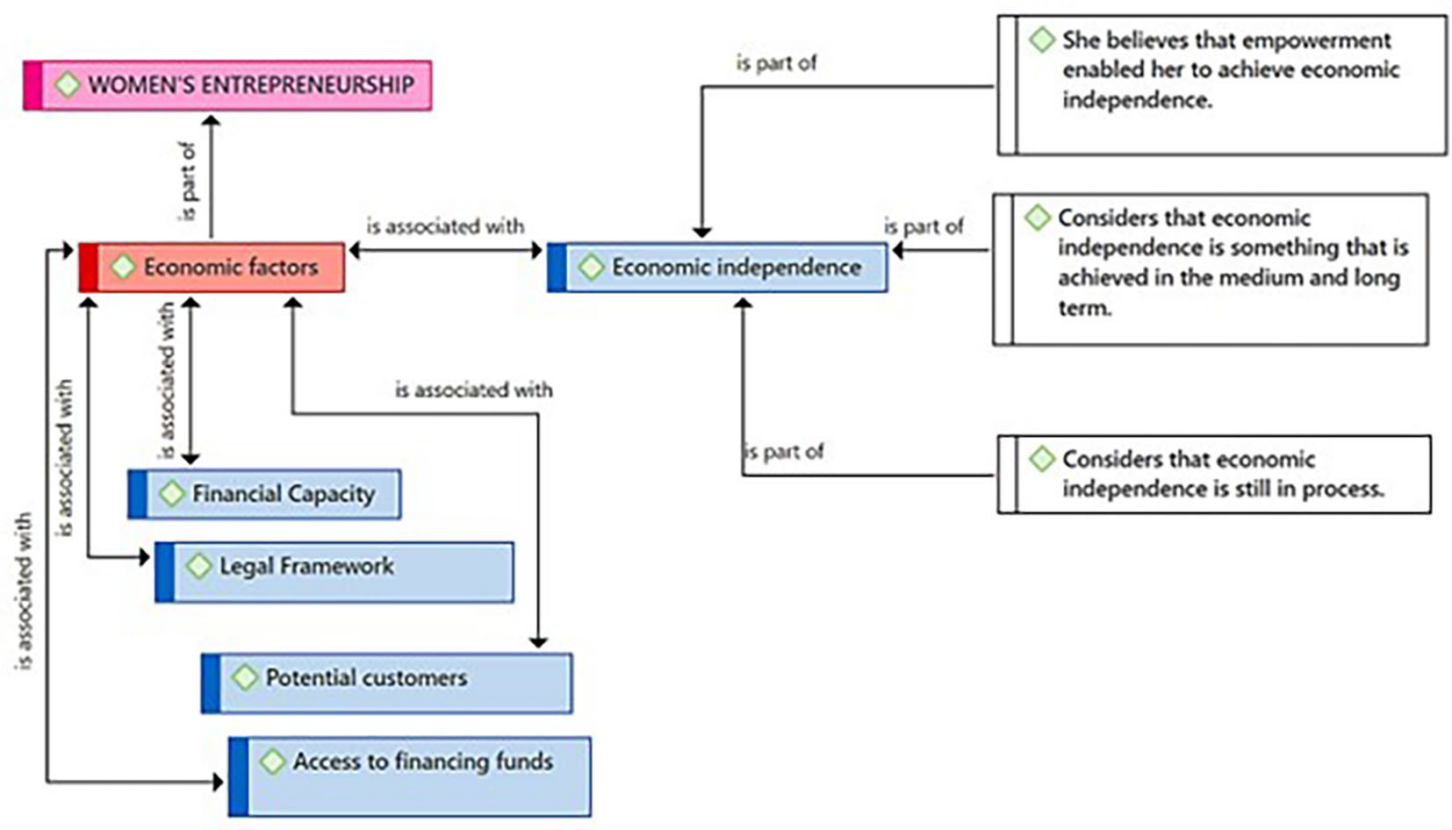
Economic Independence subcategory.

In Figure 4, the Economic Independence subcategory is highlighted as an element related to the challenges and goals of female entrepreneurship. According to the participants, empowerment is a factor that has allowed them to achieve economic independence, which implies a direct connection between personal strengthening and financial autonomy. Moreover, they consider economic independence as a medium and long-term goal, suggesting a perspective of planning and sustained development over time. Finally, they acknowledge that the process towards economic independence is ongoing, indicating that it is a dynamic and evolving state.

**Figure 5.**
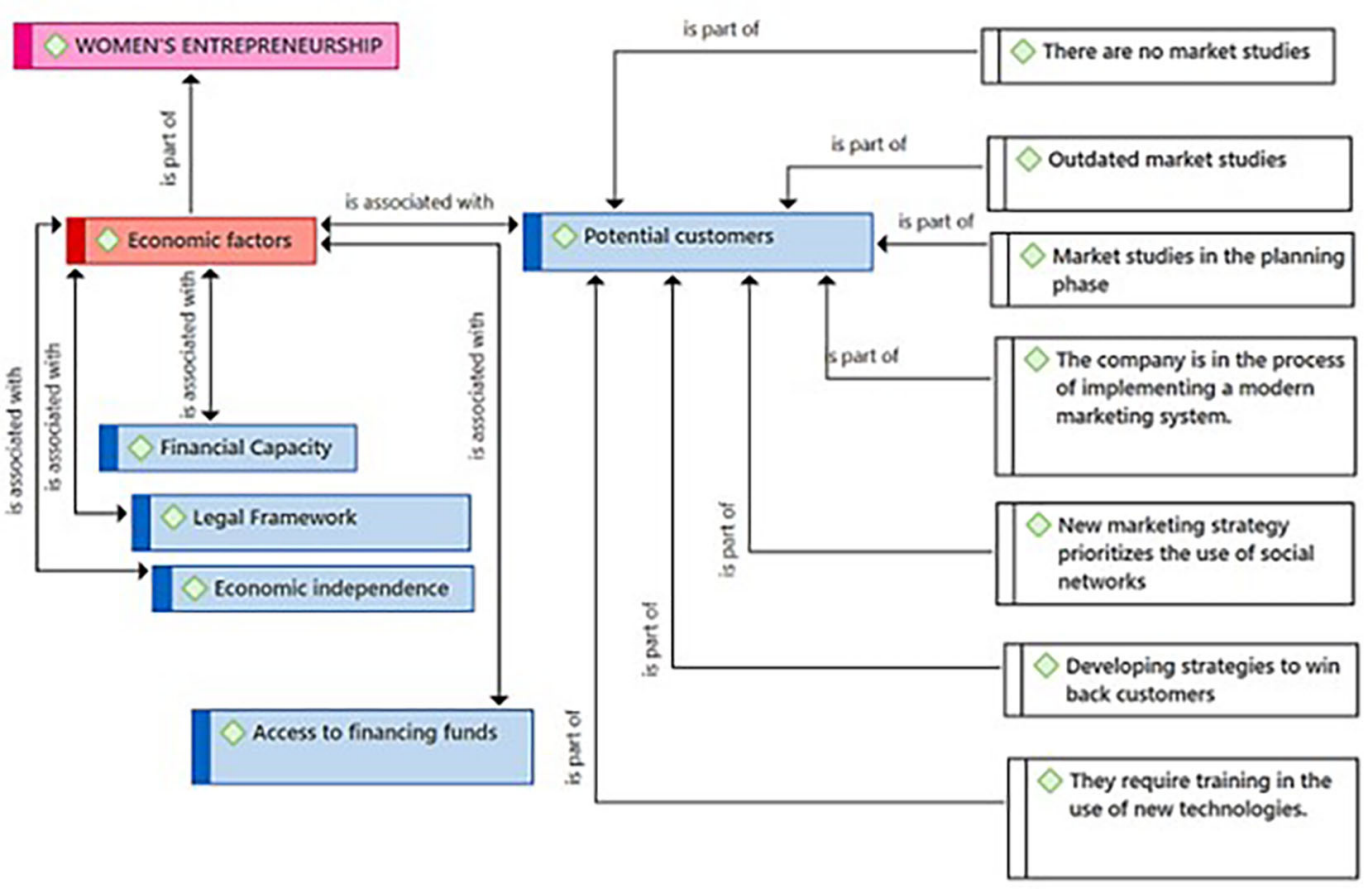
Potential Customers subcategory.

In Figure 5, the participants highlight that the lack of updated market studies and the absence of specialized studies constitute a significant limitation to fully understanding the market’s potential. Additionally, they point out that they are making efforts to gradually implement market studies that help them in the process of understanding, rescuing, and attracting new customers. They also indicate that they are in the process of implementing modern marketing systems, suggesting a transition towards modern marketing practices. They assert that the strategic and responsible use of social media is a priority for adapting to new digital marketing trends. The figure also shows that the entrepreneurs are developing strategies to recover customers, which implies a focus on customer retention and loyalty. Lastly, they identify the need for training in new technologies, thereby being able to use all the tools that allow them to reach new customers.

**Figure 6.**
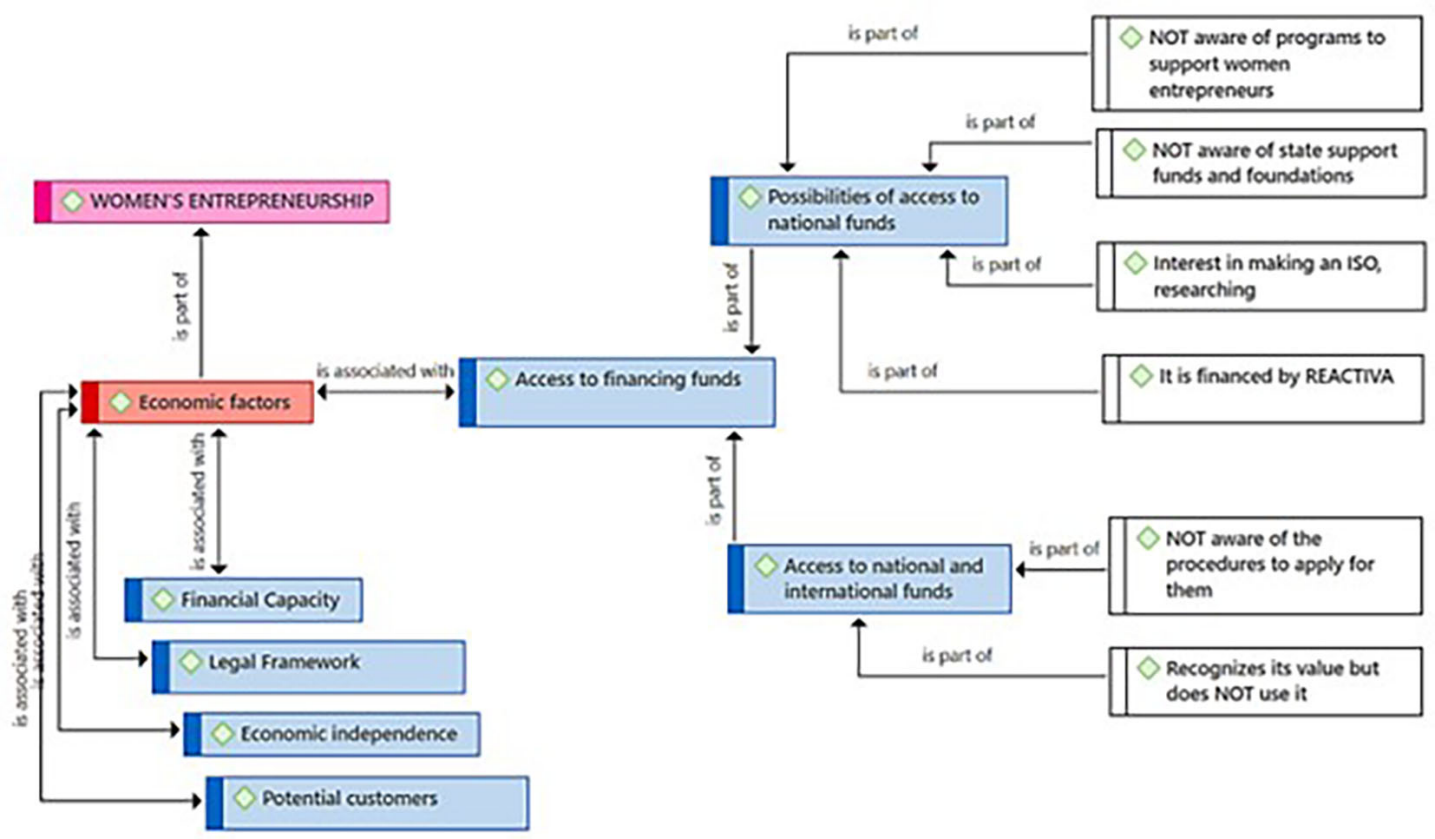
Access to Financing Funds subcategory.

Regarding the subcategory Access to Financing Funds, it presents two approaches: 1) Possibilities of access to national funds and 2) Access to national and international funds. Regarding national funds, the entrepreneurs recognize a lack of knowledge about support programs for women entrepreneurs and state support funds and foundations ( Figure 6). They also express an interest in using financial instruments such as some ISO standards, which help improve management and thereby quality. Some participants claim to have financing granted by REACTIVA-Peru (A program of credits with governmental guarantee, designed to prevent a breakdown in the payment chain). Related to the access to international funds, there is a lack of knowledge about the procedures to apply for them (this also happens regarding the access to national funds). Despite this lack of knowledge, the value of these funds is recognized, even though they cannot use them.

## 4. Discussion

According to the narrative of the participants, Financial Capacity is fundamental for the empowerment of Peruvian women, highlighting the need for funds for contingencies, access to financing, and proper management of these resources. When contrasting these results with previous research, we find, for example, a study in Ethiopia that highlights the relevance of access to credit and ownership of resources in women’s empowerment in rural areas, ^1^ while research in Murcia, Spain, shows gender differences in financing negotiation, suggesting the need to overcome additional barriers for women’s economic empowerment through a supportive environment. ^27^ Moreover, the negative cultural perception towards indebtedness in Mexico highlights the importance of addressing cultural and personal barriers to financing, an aspect that is also critical in Peru where fund management and access to financing are essential, but critical. ^28^ Similarly, studies in Mexico and Paraguay emphasize the need to improve financial capacity and access to funds as key elements for empowerment, underlining the importance of financial resources and support for entrepreneurial initiatives. ^18, 29^ Returning to Peru, other studies highlight work experience, the creation of a credit history, and governmental and non-governmental support as facilitators of access to financing; emphasizing the need for policies and programs that improve this access and promote a culture of financial management, aligned with the sustainable development goals of the 2030 agenda. ^30, 31^ These findings indicate that, despite existing challenges regarding financial capacity and access to resources, key opportunities for progress coexist through financial education, social support, and inclusive policies, underlining the importance of an integrated approach to promote women’s empowerment in Peru and enhance their economic capacity. This analysis agrees with a study conducted in the rural town of Chinchero (Cusco, Peru), where it was observed that, through entrepreneurial initiatives, businesswomen not only improved their linguistic skills (in English and Spanish) and public communication, essential for marketing their handicraft products but also generated income that significantly contributed to the success of their businesses. ^32^

Regarding the Legal Framework, participants identify as a critical component both the formalization of businesses and their alignment with legal regulations; entrepreneurs emphasize the need to adhere to the Régimen MYPE-Peru as an essential step for the development and sustainability of their ventures. This emphasis on legality and financial viability demonstrates how an adequate legal framework is vital for transparency and financial health, being crucial for the sustainability of female entrepreneurship. Research conducted in Ethiopia and Spain highlights the importance of the legal and regulatory environment in women’s economic empowerment, underscoring both the facilitations and additional barriers imposed by gender differences in financing negotiation. ^1, 27^ Similarly, studies in Mexico highlight the importance of credit programs and financial support, aligning with the need for a legal framework that promotes access to resources for entrepreneurs. ^28, 33^ These investigations converge on the urgency of policies and legal reforms that address the needs of female entrepreneurship in a more objective manner, overcoming barriers and promoting an inclusive and equitable environment. In Peru, despite having a legal framework and public policies designed to support female entrepreneurship and promote their economic empowerment, ^9– 34^ significant challenges persist that limit the full utilization of these initiatives. Therefore, the urgent need for more effective implementation and greater dissemination of these laws and strategies is crucial to overcoming the structural and cultural barriers that women entrepreneurs in the country still face. It is important to continue working on promoting a legal and social environment that not only recognizes the rights and capabilities of women but also actively facilitates their equitable participation in the economy; considering that the integration and ongoing support of women’s economic empowerment within the legal framework and public policies are fundamental to achieving a more just and equitable society.

For the participants, Economic Independence emerges as a fundamental pillar of women’s empowerment in Peru, reflecting an ongoing process of personal strengthening and financial autonomy aligned with globally observed patterns, where women’s empowerment is closely linked to economic and social factors, such as access to credit and ownership of resources. ^1^ This focus on economic independence highlights the importance of planning and sustained development towards greater autonomy, underlining the connection between economic empowerment and financial independence, especially through the implementation of credit programs aimed at women, which have proven to be crucial for the development of micro-enterprises and female productive groups. ^29, 33^ Additionally, consulted studies emphasize gender differences in financing negotiation, which directly impacts women’s economic independence, suggesting the need for policies that promote equitable access to financial resources. ^28, 35, 36^ In Peru, the need for special policies to finance ventures reflects an understanding of the challenges and opportunities to achieve economic independence, ^31^ highlighting the importance of solid financial education and support from a conducive environment that includes family, government, private banking, and NGOs. Moreover, the interconnection between economic empowerment, financial capacity, and economic independence suggests that strengthening one contributes to the advancement of the others, highlighting a global issue that requires legal reforms and public policies focused on addressing the specific needs of female entrepreneurship.

Regarding the identification and capture of Potential Customers, for the participants, it represents a significant challenge, expressing concern about the lack of updated market studies and the adoption of modern marketing strategies. In other words, most of the participants have not conducted market studies or do not have the funds to hire companies that can assist in this endeavor. Similarly, they are unaware of how to conduct such a study; therefore, the success of their ventures is usually the result of their empiricism. In this sense, the transition towards modern marketing practices, especially the strategic use of social networks and digital marketing, reflects the need to adapt to new trends to attract and retain customers, implying not only a technological change but also a shift towards a more analytical and market data-based approach. This process highlights the importance of training in new technologies to leverage all available tools to reach new customers, resonating with the importance of education and training in women’s economic empowerment. ^1^ Moreover, the strategy of offering exclusive products and relying on good taste to attract customers emphasizes the need for market differentiation to establish a distinctive brand. ^28^ The creation of social micro-enterprises and participation in productive groups point to a focus on specific markets, underlining the importance of precise market orientation and adaptation to customer needs. ^33^ Adapting to market opportunities and developing skills in marketing and research are identified as crucial for business success, ^29^ highlighting the need for additional support for some entrepreneurs in areas of limited experience and resources. This analysis suggests that success in identifying and attracting potential customers depends on the ability to adapt to market trends, product differentiation, the development of a solid brand, and the effective implementation of digital marketing strategies, in addition to emphasizing ongoing education and training in new technologies and market practices as key elements for women’s economic empowerment and business sustainability.

Access to Financing Funds is highlighted as a critical situation towards economic empowerment and business success for women, showing a key distinction between the possibilities of access to both national and international funds. The knowledge gap regarding specific support programs for women entrepreneurs and the interest in using financial instruments to improve management and quality of businesses indicate a critical need for information and accessible resources for entrepreneurs. The REACTIVA-Peru initiative, for example, underlines the existence of governmental support as it aims to “promote the financing of the replenishment of working capital funds for companies facing short-term payments and obligations with their workers and suppliers of goods and services, in order to ensure continuity in the payment chain in the national economy” ^37^; but the general lack of knowledge about how to apply for funds highlights the importance of financial education and training, to achieve a positive impact on women’s economic empowerment. ^1, 29^ On the other hand, cultural barriers to indebtedness and challenges in accessing credit, especially in rural areas, ^28^ along with the relevance of credit programs directed at women, underscore the need for financing mechanisms adapted to the unique circumstances of entrepreneurs. ^33^ In summary, the strategy of financial self-sufficiency, based on personal savings and family support, highlights the vulnerability in the absence of significant governmental support, ^35^ pointing out the need for formal financing options that support the growth and sustainability of women-led businesses. Also, the inclination towards standards, such as ISO, suggests a focus on sustainable and quality business practices, potentially increasing eligibility for funds that value high standards. Overcoming these challenges requires policies and programs that improve knowledge about available financing, offer financial education, and promote support networks to facilitate women’s economic empowerment. Finally, access to financing demands an integrated approach that includes financial education, knowledge of financing options, and the creation of a more inclusive environment for women entrepreneurs, with the collaboration of government, private sector, and NGOs to develop a business ecosystem that supports the start and sustainable growth of women’s ventures.

### 4.1 Contributions and strengths of the study

This study stands out for its rigorous hermeneutic phenomenological methodology, which allowed for a deep and meaningful exploration of the experiences of women entrepreneurs in Peru, revealing the complex influence of economic factors on their ventures. The research is characterized by a comprehensive approach, covering key categories and subcategories such as Financial Capacity, Legal Framework, Economic Independence, Potential Customers, and Access to Funds, providing a holistic and detailed perspective of the challenges and opportunities faced by women entrepreneurs. The careful selection of twelve participants, all women who have launched projects in the last five years, ensured the relevance and timeliness of the shared experiences, significantly enriching the study’s findings. Additionally, the sampling saturation criterion was effectively applied to determine the optimal number of participants, thus ensuring that the research was exhaustive and faithfully representative of the studied population, achieving a balance between depth of analysis and applicability of the results.

### 4.2 Reliability and limitations of findings

This study provides essential insights into the economic empowerment of women entrepreneurs in Peru, highlighting the need for cautious interpretation of its findings due to certain inherent limitations. Conducted in a specific region of Peru, the results offer valuable, yet contextually limited understanding, suggesting that extrapolation to other contexts or regions should be approached with caution. While the qualitative approach lends rich depth to personal narratives, the nature of this methodology implies that generalizing these findings to a broader population should be done with consideration. Additionally, although the study offers a detailed analysis focused on economic factors through twelve entrepreneurs, the future integration of non-economic variables could further enrich the understanding of female empowerment.

## 5. Conclusions

Financial Capacity is a critical subcategory for women’s economic empowerment, so the ability to adequately manage financial resources, access financing, and have funds for contingencies is fundamental for the success and sustainability of the ventures led by the participants. Likewise, promoting financial education and facilitating access to various financing sources can significantly increase the capacity to make informed economic decisions and strengthen the participants’ economic independence.

A solid Legal Framework is conducive and basic for the economic empowerment of female entrepreneurs as it facilitates the formalization of ventures, guarantees equitable rights, and promotes a safe business environment. This translates into access to financial and market benefits and is key for the transparency and financial health of female businesses. Therefore, it is necessary to implement and promote new inclusive policies that support gender equality and eliminate legal barriers that may limit female entrepreneurship.

Regarding Economic Independence, the participants perceive it as a pillar of female empowerment, allowing them to have control over their lives and economic decisions. In this sense, through entrepreneurship, participants not only improve their financial situation but also their self-esteem and participation in society. Likewise, it is crucial to support the development of entrepreneurial skills, facilitate access to markets, and promote environments that enhance the participants’ economic independence.

Identifying and managing Potential Customers is fundamental for the growth of any business. In this sense, entrepreneurs must overcome challenges such as the lack of market studies and adapt to modern marketing strategies, especially in the digital age. Therefore, receiving training in marketing, market studies, and promoting the use of technologies to improve understanding and capture of customers can significantly boost female business success.

Finally, Access to National and International Funds is viewed as a significant obstacle to the development and sustainability of ventures. Therefore, it is necessary to increase awareness, knowledge, and application to available support programs and simplifying application procedures can improve access to funds. Additionally, it is vital to create new inclusive financing programs that consider the specific needs of women, promoting equal opportunities in accessing financial resources for the development and expansion of their ventures.

## Ethics and consent

The research project was approved by Vice-Chancellor for Research Resolution No. 155-2023-VI-UCV. Subsequently, the Ethics Committee in Research of the School of Administration at Universidad César Vallejo reviewed the project under the review code 2024-1-36 and granted a favorable opinion on January 26, 2024.

Written Informed consent was obtained from all participating entrepreneurs.

## Data Availability

The transcription or recording of the twelve in-depth interviews cannot be publicly shared as it compromises the identity and privacy of the participants. This study is aligned with the Sustainable Development Goals, specifically in relation to gender equality and poverty eradication, ^18^ and our commitment to the integrity and well-being of the participants is paramount. The interviews contain identifiable information, such as, physical characteristics, and sensitive personal details of the entrepreneurs, making de-identification impossible without violating the confidentiality guaranteed to the participants. This commitment was approved by the Ethics Committee in Research at Universidad César Vallejo (review code: 2024-1-36), ^22^ in accordance with the principles set forth in the Declaration of Helsinki, ^19, 20^ ensuring the protection of identity and respect for the rights of the participants. Any request for access to the interview transcriptions or recordings must be formally submitted via email to ereynosa@ucv.edu.pe. The relevance of such requests will be evaluated by the research team in consultation with the Ethics Committee to ensure the privacy of the participants is upheld. Zenodo: Economic Factors Influencing the Empowerment of Peruvian Women. https://zenodo.org/doi/10.5281/zenodo.10884402 [Version 1, 2, 3] ^38^ This project contains the following extended data:
• Interview (English version).pdf • Instrument Validation Sheet_Expert 1. (ENGLISH VERSION).pdf • Instrument Validation Sheet_Expert 2. (ENGLISH VERSION).pdf • Instrument Validation Sheet_Expert 3. (ENGLISH VERSION).pdf • Instrument Validation Sheet_Expert 4. (ENGLISH VERSION).pdf • Instrument Validation Sheet_Expert 5. (ENGLISH VERSION).pdf • SRQR_checklist (Economic factors influencing the empowerment of Peruvian women).pdf Data are available under the terms of the Creative Commons Attribution 4.0 International license (CC-BY 4.0).

## Software availability statement

The attached file (Information Process File (ATLAS.ti).zip) ^38^ can be explored using ATLAS.ti 24. This software has a free version that can be accessed on the provider’s website. To access this version, go to the following link: https://goo.su/b14cVk, and on this website, select the option “Try ATLAS.ti for free”.
