# Supplementary material for: Economic factors influencing the empowerment of Peruvian women
Source: F1000Res. 2025 Mar 21;13:1182. Originally published 2024 Oct 9. [Version 2] doi: 10.12688/f1000research.149817.2 (PMC11971627; doi:10.12688/f1000research.149817.2)
Supplement: Peer review history

# Reviewer report: Reviewer response for version 2

**DOI:** 10.5256/f1000research.178837.r372154

**Published:** 2025-04-04

**Authors:** Jane Kerubo-Onsongo

**Affiliations:** 1 Kirinyaga University, Kerugoya, Kirinyaga County, Kenya

The article has been revised based on the comments made after first review.

Is the work clearly and accurately presented and does it cite the current literature?

Yes

If applicable, is the statistical analysis and its interpretation appropriate?

Not applicable

Are all the source data underlying the results available to ensure full reproducibility?

No source data required

Is the study design appropriate and is the work technically sound?

Yes

Are the conclusions drawn adequately supported by the results?

Yes

Are sufficient details of methods and analysis provided to allow replication by others?

Partly

Reviewer Expertise:

Gender equality and equity, Women  Empowerment, Higher Education Leadership and Management, Poverty reduction, Leadership and Integrity, Diplomacy and International Relations

I confirm that I have read this submission and believe that I have an appropriate level of expertise to confirm that it is of an acceptable scientific standard.

# Reviewer report: Reviewer response for version 2

**DOI:** 10.5256/f1000research.178837.r372153

**Published:** 2025-03-22

**Authors:** Nicolas Sumba Nacipucha

**Affiliations:** 1 Universidad Politécnica Salesiana Ecuador, Guayaquil, Ecuador

The article meets the quality criteria to be indexed in the journal

Is the work clearly and accurately presented and does it cite the current literature?

Yes

If applicable, is the statistical analysis and its interpretation appropriate?

Not applicable

Are all the source data underlying the results available to ensure full reproducibility?

No source data required

Is the study design appropriate and is the work technically sound?

Yes

Are the conclusions drawn adequately supported by the results?

Yes

Are sufficient details of methods and analysis provided to allow replication by others?

Yes

Reviewer Expertise:

Behavioral Economics, Marketing, Business Management

I confirm that I have read this submission and believe that I have an appropriate level of expertise to confirm that it is of an acceptable scientific standard.

# Reviewer report: Reviewer response for version 1

**DOI:** 10.5256/f1000research.164323.r352292

**Published:** 2025-01-02

**Authors:** Nicolas Sumba Nacipucha

**Affiliations:** 1 Universidad Politécnica Salesiana Ecuador, Guayaquil, Ecuador

The article offers an insightful exploration of the economic factors impacting the empowerment of women entrepreneurs in northern Peru, employing a hermeneutic phenomenological methodology. A notable aspect of this work is its comprehensive approach, which includes key categories such as Financial Capacity, Legal Framework, Economic Independence, Potential Customers, and Access to Funds, providing a holistic perspective on the challenges and opportunities of female entrepreneurship in this Latin American country.

 The conclusions are clear and relevant, emphasizing the importance of financial education, legal formalization, access to inclusive financing, and training in market strategies and technology—elements that strengthen the economic autonomy and social impact of the participants.

 The study's findings are particularly valuable for Peruvian authorities (as well as for authorities in neighboring countries such as Ecuador and Bolivia) to take action and design strategies that bolster the entrepreneurial efforts of their female citizens.

Is the work clearly and accurately presented and does it cite the current literature?

Yes

If applicable, is the statistical analysis and its interpretation appropriate?

Not applicable

Are all the source data underlying the results available to ensure full reproducibility?

No source data required

Is the study design appropriate and is the work technically sound?

Yes

Are the conclusions drawn adequately supported by the results?

Yes

Are sufficient details of methods and analysis provided to allow replication by others?

Yes

Reviewer Expertise:

Behavioral Economics, Marketing, Business Management

I confirm that I have read this submission and believe that I have an appropriate level of expertise to confirm that it is of an acceptable scientific standard.

# Reviewer report: Reviewer response for version 1

**DOI:** 10.5256/f1000research.164323.r334862

**Published:** 2024-11-08

**Authors:** Jane Kerubo-Onsongo

**Affiliations:** 1 Kirinyaga University, Kerugoya, Kirinyaga County, Kenya

This article reports on research findings on  factors that  contribute to the economic empowerment of female entrepreneurs in Northern Peru. The findings  reveal that in spite of the legal framework there are challenges that face women that need attention by policy makes and researchers. 

 The article would be strengthened if more information would be provided about the context. For example

 What is the population of women entrepreneurs compared to men in Northern Peru. The authors justify the selection of the region because  "female entrepreneurs in this region is has begun to stand out significantly in recent years? What has been the situation? How are they standing out? Is it that the number of business ( SMES) operated to women compared to men has increased or are they making more profits?. What is the gender inequality/inequity in Peru? How about poverty Index? Are women poorer than Men? Which targets of the SDG 1 & 5 does your study/findings contribute to?

 Sample

 What types of enterprises were the twelve female entrepreneurs selected engaged in. Is it assumed that the challenges faces by women entrepreneurs are similar regardless of the enterprise?

 Was there any attempt to triangulate the data with other sources like Government records on financial supports to female entrepreneurs'?

Is the work clearly and accurately presented and does it cite the current literature?

Yes

If applicable, is the statistical analysis and its interpretation appropriate?

Not applicable

Are all the source data underlying the results available to ensure full reproducibility?

No source data required

Is the study design appropriate and is the work technically sound?

Yes

Are the conclusions drawn adequately supported by the results?

Yes

Are sufficient details of methods and analysis provided to allow replication by others?

Partly

Reviewer Expertise:

Gender equality and equity, Women  Empowerment, Higher Education Leadership and Management, Poverty reduction, Leadership and Integrity, Diplomacy and International Relations

I confirm that I have read this submission and believe that I have an appropriate level of expertise to confirm that it is of an acceptable scientific standard, however I have significant reservations, as outlined above.
